# Supplementary material for: Accurate timekeeping is controlled by a cycling activator in Arabidopsis
Source: eLife. 2013 Apr 30;2:e00473. doi: 10.7554/eLife.00473 (PMC3639509; doi:10.7554/eLife.00473)
Supplement: Supplementary file 2. — Promoters were defined as the 1500 bp region upstream of the translational start site and motifs were identified using the SCOPE motif finder (Carlson et al., 2007). Both strands were considered for calculation of significance. Background frequency was determined using all genes in the genome. (A) Up-regulated CCGs (376 genes). (B) Down-regulated CCGs (525 genes). DOI: http://dx.doi.org/10.7554/eLife.00473.015 [file elife00473s002.doc]

**Supplementary file 2. Promoter motifs overrepresented in RVE8-regulated CCGs (related to Table 1).**

Promoters were defined as the 1500 bp region upstream of the translational start site and motifs were identified using the *SCOPE* motif finder (Carlson et al., 2007). Both strands were considered for calculation of significance. Background frequency was determined using all genes in the genome.

**(A) Up-regulated CCGs (376 genes)**

| **Motif** | **Sequence** | **Count** | **Significance^1^** | **Coverage^2^** |
| --- | --- | --- | --- | --- |
| short EE | AAATATCT | 285 | 211.7 | 49.50% |
| EE-like | AATATCT | 442 | 159.7 | 65.90% |
| long EE | AAAATATCT | 162 | 155.8 | 32.80% |

**(B) Down-regulated CCGs (525 genes)**

| **Motif** | **Sequence** | **Count** | **Significance** | **Coverage** |
| --- | --- | --- | --- | --- |
| G-box-like | BACGTRD^3^ | 1527 | 224.7 | 82.50% |
| ME-like | CCACA | 1558 | 124 | 92.20% |

^1^ Significance is the negative logarithm of an expectation.

^2^ Coverage is the percentage of the genes in the query that contain at least one copy of the consensus sequence being examined.

^3^ B = C/G/T, R = A/G, D = A/G/T
