# Supplementary material for: Accurate timekeeping is controlled by a cycling activator in Arabidopsis
Source: eLife. 2013 Apr 30;2:e00473. doi: 10.7554/eLife.00473 (PMC3639509; doi:10.7554/eLife.00473)
Supplement: Supplementary file 3. — DOI: http://dx.doi.org/10.7554/eLife.00473.016 [file elife00473s003.docx]

**Supplementary file 3. Primers used in this study.**

| **Primer** | **Sequence** | **Purpose** | **Reference** |
| --- | --- | --- | --- |
| RVE8p-SacI/NotI | GAGCTCgcggccgccgtaagatttgaatacaaaaccg | make RVE8::RVE8:GR |  |
| RVE8-GR-R | agcttcaggatccaagcttatcgatgctgatttgtcgcttgttgag | make RVE8::RVE8:GR |  |
| GR-F | tcgataagcttggatcctgaagct | make RVE8::RVE8:GR |  |
| OCS-R | cagcggccgcagatttaggtga | make RVE8::RVE8:GR |  |
| RVE8-F-XhoI | CTCGAGatgagctcgtcgccgtcaaga | make 35S::RVE8:GR |  |
| RVE8-R-SmaI | CCCGGGgttgctgatttgtcgcttgttgag | make 35S::RVE8:GR |  |
| RVE4-F | TCGTCGTCATTTCTCTCTCTAACC | genotyping |  |
| RVE4-R | AACAGGCACAGGATGATTGAGTGG | genotyping |  |
| LB b1 | GCGTGGACCGCTTGCTGCAACT | genotyping | (Alonso et al., 2003) |
| RVE6-F | GCGTGGAGTTTCGATTAGGATAATG | genotyping |  |
| RVE6-R | CCATTCTTGTGAGCCTTCTGAGG | genotyping |  |
| RVE8-F | caccatgagctcgtcgccgtcaagaaa | genotyping |  |
| RVE8-R | TGTTTAGCAGCATTGAGGCATCATC | genotyping |  |
| RVE4 qPCR-F | GTGGTCGCCGAAGTAATACC | qPCR |  |
| RVE4 qPCR-R | TCTGGTGGAATAACCCCACTT | qPCR |  |
| RVE6 qPCR-F | ACGGAGCCTGAGCATGATAAATTCC | qPCR |  |
| RVE6 qPCR-R | TGAATGACCCTGGTACTTGCAGTTG | qPCR |  |
| RVE8 qPCR-F | GGGAAGCTCAAGCCGAACAGTATC | qPCR | (Rawat et al., 2011) |
| RVE8 qPCR-R | GGCCTCTCGTTTCAGGATCAAAGA | qPCR | (Rawat et al., 2011) |
| GI qPCR-F | ACTAGCAGTGGTCGACGGTTTATC | qPCR | (Mockler et al., 2004) |
| GI qPCR-R | GCTGGTAGACGACACTTCAATAGATT | qPCR | (Mockler et al., 2004) |
| TOC1qPCR-F | AATAGTAATCCAGCGCAATTTTCTTC | qPCR | (Mockler et al., 2004) |
| TOC1qPCR-R | CTTCAATCTACTTTTCTTCGGTGCT | qPCR | (Mockler et al., 2004) |
| LHY qPCR-F | CAATGCAACTACTGATTCGTGGAA | qPCR | (Mockler et al., 2004) |
| LHY qPCR-R | GCTATACGACCCTCTTCGGAGAC | qPCR | (Mockler et al., 2004) |
| CCA1 qPCR-F | CAGCTCCAATATAACCGATCCAT | qPCR | (Mockler et al., 2004) |
| CCA1 qPCR-R | CAATTCGACCCTCGTCAGACA | qPCR | (Mockler et al., 2004) |
| CCR2 qPCR-F | CTTGATCTTCCAGTCTCACGAT | qPCR | (Martin-Tryon et al., 2007) |
| CCR2 qPCR-R | CGACGTTATTGATTCCAAGATCA | qPCR | (Martin-Tryon et al., 2007) |
| PRR5 qPCR-F | ATTCCGAATGAAGCGAAAGGA | qPCR | (Mockler et al., 2004) |
| PRR5 qPCR-R | TCGTAACGAACCTTTTTCTCATAACAT | qPCR | (Mockler et al., 2004) |
| PRR9 qPCR-F | GTTGAAGAGGAAAGATCGATGCTT | qPCR | (Mockler et al., 2004) |
| PRR9 qPCR-R | CTGCTCTGGTACCGAACCTTTT | qPCR | (Mockler et al., 2004) |
| PP2A-F qPCR | TAACGTGGCCAAAATGATGC | qPCR | (Czechowski et al., 2005) |
| PP2A-R qPCR | GTTCTCCACAACCGATTGGT | qPCR | (Czechowski et al., 2005) |
| ELF4-F qPCR | CAAAGCAACGTTCTTCGACA | qPCR | (Martin-Tryon and Harmer, 2008) |
| ELF4-R qPCR | CGACAATCACCAATCGAGAA | qPCR | (Martin-Tryon and Harmer, 2008) |
| LUX-F qPCR | CGGATTCGAAGAAGCAAAAG | qPCR | (Martin-Tryon and Harmer, 2008) |
| LUX-R qPCR | TCATCTCCATCACCGTTTGA | qPCR | (Martin-Tryon and Harmer, 2008) |
| 5G17220 qPCR-F | TCTTCTTCGTCAGCCATTTGGTC | qPCR | (Su et al., 2011) |
| 5G17220 qPCR-R | GGGCCAGAACGTTGAAGTAATAGG | qPCR | (Su et al., 2011) |
| wtCCR2_EE_F | GAGGTCAAACCTAGAAAATATCTAAACCTTGAAACCTAG | EMSA | (Harmer and Kay, 2005) |
| wtCCR2_EE_R | GGAGCTAGGTTTCAAGGTTTAGATATTTTCTAGGTTTGA | EMSA | (Harmer and Kay, 2005) |
| muCCR2_EE_F | GAGGTCAAACCTAGAAAATCGAGAAACCTTGAAACCTAG | EMSA | (Harmer and Kay, 2005) |
| muCCR2_EE_R | GGAGCTAGGTTTCAAGGTTTCTCGATTTTCTAGGTTTGA | EMSA | (Harmer and Kay, 2005) |
| PE-1 | AATGATACGGCGACCACCGAGATCTACACTCTTTCCCTACACGACGCTCTTCCGATCT | library amplification | Illumina |
| PE-2 | CAAGCAGAAGACGGCATACGAGATCGGTCTCGGCATTCCTGCTGAACCGCTCTTCCGATCT | library amplification | Illumina |
| PE1-AAGAC | GTCTTAGATCGGAAGAGCGGTTCAGCAGGAATGCCGAG | indexed adaptor |  |
| PE1-ACCAT | ATGGTAGATCGGAAGAGCGGTTCAGCAGGAATGCCGAG | indexed adaptor |  |
| PE1-CATTA | TAATGAGATCGGAAGAGCGGTTCAGCAGGAATGCCGAG | indexed adaptor |  |
| PE1-GTAGG | CCTACAGATCGGAAGAGCGGTTCAGCAGGAATGCCGAG | indexed adaptor |  |
| PE1-TGCCT | AGGCAAGATCGGAAGAGCGGTTCAGCAGGAATGCCGAG | indexed adaptor |  |
| PE1-TGGTA | TACCAAGATCGGAAGAGCGGTTCAGCAGGAATGCCGAG | indexed adaptor |  |
| PE1-AACCG | CGGTTAGATCGGAAGAGCGGTTCAGCAGGAATGCCGAG | indexed adaptor |  |
| PE1-CTGGA | TCCAGAGATCGGAAGAGCGGTTCAGCAGGAATGCCGAG | indexed adaptor |  |
| PE1-TGATC | GATCAAGATCGGAAGAGCGGTTCAGCAGGAATGCCGAG | indexed adaptor |  |
| PE1-GCTAT | ATAGCAGATCGGAAGAGCGGTTCAGCAGGAATGCCGAG | indexed adaptor |  |
| PE1-CCAGC | GCTGGAGATCGGAAGAGCGGTTCAGCAGGAATGCCGAG | indexed adaptor |  |
| PE1-GTTCG | CGAACAGATCGGAAGAGCGGTTCAGCAGGAATGCCGAG | indexed adaptor |  |
| PE2-AAGAC | ACACTCTTTCCCTACACGACGCTCTTCCGATCTAAGACT | indexed adaptor |  |
| PE2-ACCAT | ACACTCTTTCCCTACACGACGCTCTTCCGATCTACCATT | indexed adaptor |  |
| PE2-CATTA | ACACTCTTTCCCTACACGACGCTCTTCCGATCTCATTAT | indexed adaptor |  |
| PE2-GTAGG | ACACTCTTTCCCTACACGACGCTCTTCCGATCTGTAGGT | indexed adaptor |  |
| PE2-TGCCT | ACACTCTTTCCCTACACGACGCTCTTCCGATCTTGCCTT | indexed adaptor |  |
| PE2-TGGTA | ACACTCTTTCCCTACACGACGCTCTTCCGATCTTGGTAT | indexed adaptor |  |
| PE2-AACCG | ACACTCTTTCCCTACACGACGCTCTTCCGATCTAACCGT | indexed adaptor |  |
| PE2-CTGGA | ACACTCTTTCCCTACACGACGCTCTTCCGATCTCTGGAT | indexed adaptor |  |
| PE2-TGATC | ACACTCTTTCCCTACACGACGCTCTTCCGATCTTGATCT | indexed adaptor |  |
| PE2-GCTAT | ACACTCTTTCCCTACACGACGCTCTTCCGATCTGCTATT | indexed adaptor |  |
| PE2-CCAGC | ACACTCTTTCCCTACACGACGCTCTTCCGATCTCCAGCT | indexed adaptor |  |
| PE2-GTTCG | ACACTCTTTCCCTACACGACGCTCTTCCGATCTGTTCGT | indexed adaptor |  |

References:

Alonso JM, Stepanova AN, Leisse TJ, Kim CJ, Chen H, Shinn P. 2003. Genome-wide insertional mutagenesis of Arabidopsis thaliana. Science 301:653–7. doi: 10.1126/science.1086391.

Czechowski T, Stitt M, Altmann T, Udvardi MK, Scheible WR. 2005. Genome-wide identification and testing of superior reference genes for transcript normalization in Arabidopsis. Plant Physiol 139:5–17. doi: 10.1104/pp.105.063743.

Martin-Tryon EL, Harmer SL. 2008. XAP5 CIRCADIAN TIMEKEEPER coordinates light signals for proper timing of photomorphogenesis and the circadian clock in Arabidopsis. Plant Cell 20:1244–59. doi: 10.1105/tpc.107.056655.

Martin-Tryon EL, Kreps JA, Harmer SL. 2007. GIGANTEA acts in blue light signaling and has biochemically separable roles in circadian clock and flowering time regulation. Plant Physiol 143:473–86. doi: 10.1104/pp.106.088757.

Mockler TC, Yu X, Shalitin D, Parikh D, Michael TP, Liou J. 2004. Regulation of flowering time in Arabidopsis by K homology domain proteins. Proc Natl Acad Sci USA 101:12759–64. doi: 10.1073/pnas.0404552101.

Rawat R, Takahashi N, Hsu PY, Jones MA, Schwartz J, Salemi MR. 2011. REVEILLE8 and PSEUDO-REPONSE REGULATOR5 form a negative feedback loop within the Arabidopsis circadian clock. PLoS Genet 7:e1001350. doi: 10.1371/journal.pgen.1001350.

Su T, Xu J, Li Y, Lei L, Zhao L, Yang H. 2011. Glutathione-indole-3-acetonitrile is required for camalexin biosynthesis in Arabidopsis thaliana. Plant Cell 23:364–80. doi: 10.1105/tpc.110.079145.
